# The combination of Chinese and Western Medicine in the management of rheumatoid arthritis: A real-world cohort study across China

**DOI:** 10.3389/fphar.2022.933519

**Published:** 2022-10-06

**Authors:** Linda LD. Zhong, Rongsheng Wang, Wai Ching Lam, Qi Zhu, Peipei Du, Pei Hua Cao, Ting Jiang, Yuan Yuan Zhang, Jie Shen, Xiao Su, Luan Xue, Jianchun Mao, Yong Fei Fang, Ming Li Gao, Chun Rong Hu, Jiang Yun Peng, Ying Gu, Qianghua Wei, Runyue Huang, Aiping Lyu, Hongxia Liu, Dongyi He

**Affiliations:** ^1^ School of Chinese Medicine, Hong Kong Baptist University, Hong Kong, China; ^2^ School of Biological Sciences, Nanyang Technological University, Singapore, Singapore; ^3^ Department of Rheumatology, Guangdong-Hong Kong-Macau Joint Lab on Chinese Medicine and Immune Disease Research, Hong Kong, China; ^4^ Department of Medicine, ShangHai GuangHua Hospital of Integrated Traditional Chinese and Western Medicine, Shanghai, China; ^5^ Institute of Arthritis Research in Integrative Medicine, Shanghai Academy of Traditional Chinese Medicine, Shanghai, China; ^6^ Department of Rheumatology and Immunology, Shanghai Municipal Hospital of Traditional Chinese Medicine, Shanghai, China; ^7^ Department of Rheumatology and Immunology, Yueyang Hospital of Integrated Traditional Chinese and Western Medicine, Shanghai University of Traditional Chinese Medicine, Shanghai, China; ^8^ Department of Rheumatology, LongHua Hospital Shanghai University of Traditional Chinese Medicine, Shanghai, China; ^9^ Department of Rheumatology, Southwest Hospital, Chongqing, China; ^10^ Department of Rheumatology, Liaoning Hospital of Traditional Chinese Medicine, Liaoning, China; ^11^ Department of Rheumatology, The Ninth People’s Hospital of Chongqing, Chongqing, China; ^12^ Department of Rheumatology, Yunnan Provincial Hospital of Traditional Chinese Medicine, Yunnan, China; ^13^ Department of Rheumatology, Mianyang Hospital of Traditional Chinese Medicine, Sichuan, China; ^14^ Department of Rheumatology, Shanghai General Hospital, Shanghai, China; ^15^ The Second Affiliated Hospital of Guangzhou University of Chinese Medicine (Guangdong Provincial Hospital of Chinese Medicine), Guangzhou, China; ^16^ State Key Laboratory of Dampness Syndrome of Chinese Medicine (The Second Affiliated Hospital of Guangzhou University of Chinese Medicine), Guangzhou, China; ^17^ Guangdong Provincial Key Laboratory of Clinical Research on Traditional Chinese Medicine Syndrome, Guangzhou, China; ^18^ Center for Drug Clinical Research, Shanghai University of Traditional Chinese Medicine, Shanghai, China

**Keywords:** cohort study, integrative medicine, rheumatoid arthritis, autoimmune disease, clinical study

## Abstract

**Objective:** To investigate the efficacy of Integrative medicine (IM), compare with Western medicine (WM), in the treatment of rheumatoid arthritis (RA) in a cohort study.

**Methods:** This is a cohort study with recruitment of RA patients from 10 hospitals in China. The primary outcome was change in disease activity score 28 (DAS28) during 4 follow-up visits. Generalized estimating equation (GEE) models that controlled for variables were used to investigate a time trend and assess group differences in the primary outcome and secondary outcomes after propensity score matching (PSM).

**Results:** A total of 3195 patients with RA received IM (n = 1379, 43.2%) or WM (n = 1816, 56.8%). Following 1:1 propensity score matching, 1,331 eligible patients prescribed IM were compared to 1,331 matched patients prescribed WM. The GEE analysis with PSM showed that the IM was more beneficial to significantly decrease the levels of VAS, PGA and PhGA (VAS: odds ratio (OR), 0.76; 95% CI, 0.63–0.92; *p* = 0.004; PGA: OR, 0.76; 95% CI, 0.64–0.92; *p* = 0.007; and PhGA: OR, 0.77; 95% CI, 0.64, 0.93; *p* = 0.004), and reduce DAS28 (OR, 0.84; 95% CI, 0.73–0.98; *p* = 0.030) in the per-protocol population.

**Conclusion:** This study suggests that compare to WM, IM has advantages in improving RA-related outcomes. However, the statistical significance might not reveal significant clinical difference. Further studies should be focused on specific treatment strategies and/or disease stages.

## 1 Introduction

Management of rheumatoid arthritis (RA) symptoms using Integrative medicine (IM), such as a combination of Chinese medicine (CM) and Western medicine (WM), has been widely adopted among Chinese populations (Zhao et al., 2013). With growing needs in the public and interests among investigators, number of clinical studies, including trials and reviews, have been trending up in recent decades.

Although existing clinical trials provided evidence for the efficacy and safety of integrative approaches in treating RA (Xing et al., 2020; Lu et al., 2022), the studies only involved limited kinds of interventions and cannot reflect the situations happening in the real-world. Moreover, clinical trials are not feasible given the large numbers of participants needed and high adherence with long follow-up (Zhang et al., 2015).

Currently in China, the prevalence of RA patients is estimated to be tens of million across 23 provinces (Langley et al., 2011). Rather than biologic therapies, patients are prescribed IM or WM as they visit the primary health-care system. The majority of WM prescribed covers steroids, non-steroidal anti-inflammatory drugs (NSAIDs), and disease-modifying antirheumatic drugs (DMARDs). On the other hand, the prescriptions of Chinese herbal medicine can be variable according to the CM diagnosis. While the use of IM for RA has been the norm in recent decades, there are still lack of large-scale studies to review the efficacy. An overview of whether the combined use of CM and WM could be beneficial to the prognosis of RA becomes critical.

Given the complexity in clinical settings and limited evidence on the advantages of using IM in alleviating RA related symptoms, we proposed this cohort study to investigate the efficacy of IM in the treatment of RA.

## 2 Material and methodology

### 2.1 Study design

This cohort study was conducted through the National Integrative Medicine Network for Rheumatoid Diseases from February 2014 to August 2018. The 10 hospitals in the network included I) ShangHai GuangHua Hospital of Integrated Traditional Chinese and Western Medicine, II) Mianyang Hospital of Traditional Chinese Medicine, III) The Ninth People’s Hospital of Chongqing, IV) Shanghai Municipal Hospital of Traditional Chinese Medicine, V) Yueyang Hospital of Integrated Traditional Chinese and Western Medicine, Shanghai University of Traditional Chinese Medicine, VI) Southwest Hospital, VII) LongHua Hospital Shanghai University of Traditional Chinese Medicine, VIII) Liaoning Hospital of Traditional Chinese Medicine, IX) Yunnan Provincial Hospital of Traditional Chinese Medicine, and X) Shanghai General Hospital. The study was approved by the Research Ethics Committee of ShangHai GuangHua Hospital specifically for this research (No. 2014-K-04) and the ethics approval has been circulated and endorsed by the other nine hospital ethics committees before initiating the research. Public promotion was adopted within the hospitals and affiliated outpatient clinics. Both inpatients and outpatients of Rheumatology were able to sign up for the study.

### 2.2 Participants

Eligible patients aged 18 years or older with RA for at least 3 months who fulfilled the 1987 American College of Rheumatology (ACR) or 2010 European Alliance of Associations for Rheumatology (EULAR) diagnostic criteria diagnosed by physicians (Britsemmer et al., 2011; Kay and Upchurch, 2012). Patients were excluded if they had little or were lack of ability for self-care; confusion in diagnosis caused by acute and chronic infections; been diagnosed with severe, progressive, or uncontrolled diseases on heart, liver, kidney, gastroenterology, endocrinology, hematology, or cancer; history of joint surgery; medical history of neurological diseases or psychiatric disorders; been currently participating in clinical trials. All the participants gave written informed consent. Patients included in the study received either 1) IM or 2) WM during the treatment based on clinical considerations of physicians. The IM is defined as combined treatment of WM and CM. WM consists of pharmacotherapy for RA including steroids, NSAIDs, and DMARDs. On the other hand, CM involved Chinese herbal decoctions, or tablets/capsules composed solely of Chinese herbs and their extracts. The types of medications used by patients with RA at baseline and during follow-up were shown in Table 1. All participants were then followed up 1 year with 3-month intervals: at 3 months, 6 months, 9 months, and 12 months from the baseline visit. Since there were cases with a discrepancy between the medication at each follow-up, we defined study populations as follows to delineate the genuine effect of IM or WM treatment; intention-to-treat (ITT) and per-protocol (PP) populations. The ITT population was defined by patients who received medication at baseline. The PP population was restricted to the population who received the same medication at baseline and at follow-up.

**TABLE 1:** Types of medications used by patients with Rheumatoid Arthritis at baseline and during follow-up.

Medication type	No.	%
**Steroids**		
Methylprednisolone	1328	54.99
Prednisone	1015	42.03
Others	72	2.98
**NSAIDs**		
Meloxicam	1389	32.09
Nimesulide	1012	23.38
Loxoprofen Sodium	600	13.86
Celecoxib	377	8.71
Diclofenac	350	8.09
Others	610	14.09
**DMARDs**		
Methotrexate	4647	46.00
Leflunomide	2484	24.59
Penicillamine	808	8.00
Hydroxychloroquine	668	6.61
Salazosulfadimidine	393	3.89
Iguratimod	325	3.22
Hydroxychloroquine Sulfate	232	2.30
Others	545	5.39
**Chinese Medicine products**		
Tripterygium Glycoside	650	19.63
Zhengqinfengtongning	610	18.42
Total Glucosides of Paeonia	583	17.61
Zaocys Dhumnades Preparations	299	9.03
Kunxian Capsule	261	7.88
Biqi Capsule	107	3.23
Girald Daphne Bark	106	3.20
Yishen Juanbi Pill	92	2.78
Others	603	18.21

### 2.3 Study variables and propensity score matching

The following baseline variables were gathered from designed questionnaire: patient demographic characteristics (age, gender, body mass index (BMI)), family history of rheumatic immunity (RI)-related, operation history of RI-related, smoking, alcohol use, comorbidities (hypertension, diabetes, et al.), duration of RA.

To reduce the potential biases in selection and lost to follow-up, we used propensity score matching (PSM) to match the comparative groups in ITT and PP population. Propensity scores for the type of medicine were estimated using a logistic regression model including all the above-mentioned baseline variables. Participants in the IM group were matched 1:1 to the WM group, by propensity score using the nearest neighbor algorithm, with a caliper width of 0.05.

### 2.4 Outcome measures

The primary outcome was change in disease activity score 28 (DAS28) during 4 follow-up visits. The secondary outcomes included change in tender joint count (TJC), swollen joint count (SJC), morning stiffness (MS), visual analog scale (VAS), patient’s and physician’s global assessment of disease activity based on visual analogue scale (PGA, PhGA), erythrocyte sedimentation rate (ESR), c-reactive protein (CRP), rheumatoid factor (RF), anti-cyclic citrullinated peptide (Anti-CCP), simplified disease activity index (SDAI), clinical disease activity index (CDAI), health assessment questionnaire (HAQ) during 4 follow-up visits.

### 2.5 Statistical analysis

Continuous variables were expressed as mean (standard deviation) or medians (interquartile range, IQR), depending on the data distribution pattern. Categorical variables were described using frequencies and percentages. The Multiple Imputation (MI) by chained equations method was used to interpolate the missing data. Baseline characteristics before and after PSM were compared between IM and WM groups using the variance analysis Kruskal–Wallis rank-sum test for continuous variables and Chi-square test for categorical variables. Generalized estimating equation (GEE) models that controlled for variables were used to investigate a time trend and assess group differences in the primary outcome and secondary outcomes after PSM. This was done using a GEE autoregressive time-lag model that correlates the IM or WM on RA related clinical outcomes 1 year later. The IM or WM was used as an independent variable, each RA related clinical outcomes at baseline was used as control variable, and the corresponding continuous clinical outcomes were used as dependent variables. Significance levels were set at a 2-tailed *p* < 0.05. Statistical analyses were performed using R (Version 4.1.0).

## 3 Results

**FIGURE 1:**
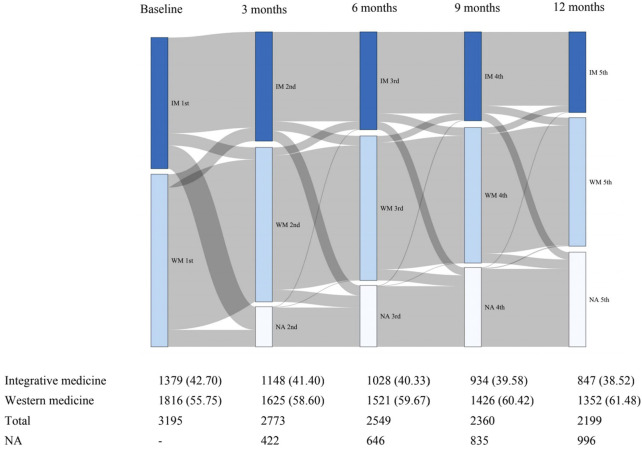
Sankey diagram of changes in clinical medicine during the first year of follow-up. IM: Integrative medicine, WM: Western medicine.

A total of 3195 patients with RA received IM (n = 1379, 43.2%) or WM (n = 1816, 56.8%) and were included in the ITT population (Figure 1). During the 1-year observational period, 996 patients lost to follow up, and a comparison of baseline characteristics between the lost and follow-up groups was shown in Supplementary Table S1. 1619 (50.7%) patients continued the same medication until 12 months and were considered as the PP population. During the 1-year follow-up, a total of 1576 ITT patients changed the type of medication from WM to IM or vis versa. The comparison of baseline characteristics between the unchanged medication group and the medication changed group was shown in Supplementary Table S2. The frequency of missing baseline information of the unchanged medication patients was 38 (1.19%) cells (Supplementary Table S3). Throughout the study, we recorded abnormal values from the patients based on their laboratory test results. However, we are not able to judge whether the abnormalities were caused by the disease or/and the treatments. In the study, no serious adverse event was observed. There was no direct evidence showing a significant difference in the rate of self-reporting adverse events in the PP population between the two groups (*p* = 0.713), which was 1.35% and 1.72% in the WM and IM groups, respectively.

In the unmatched groups (1,816 patients treated by WM compared to 1,379 patients treated by IM), the IM group was older (mean age 62.02 versus 60.34 years, *p* < 0.001) and had a higher level of BMI (22.09 versus 21.89 kg/m2, *p* = 0.036) compared to the WM group in the ITT population (Supplementary Table S4). Compared to the WM group, the IM group had similar proportion of males, family history and operation history of RI-related. The IM group had higher rates of smoking and drinking. 256 (18.6%) patients reported at least one comorbidity in the IM group which was slightly higher than the WM group (*p* = 0.044). However, the prevalence of hypertension and diabetes was similar in both groups. The median duration of RA of both groups was about 6 years.

Following 1:1 propensity score matching, 1,331 eligible patients prescribed IM were compared to 1,331 matched patients prescribed WM. After matching, the demographic and clinical characteristics of both groups were well balanced (Supplementary Table S4).

Comparisons of treatment groups in the PP population before PSM showed that the IM group were older than the WM group (mean age 64.66 versus 60.96 years, *p* < 0.001). The proportions of male patients were about 19% in both groups. 56 (5.4%) patients in the WM group had family history of RI-related which was higher than the IM group (*p* = 0.018). Patients in the WM group had higher smoking rates (2.4% versus 1.0%, *p* = 0.050). 553 eligible patients prescribed IM were compared to 553 matched patients prescribed WM after PSM. No statistically significant differences were found in baseline variables after matching between groups (Table 2).

**TABLE 2:** Patient characteristics before and after PSM in PP population.

Demographics	Unmatched (n = 1619)		After PSM (n = 1106)			
	WM (n = 1036)	IM (n = 583)	*p* value^a^	WM (n = 553)	IM (n = 553)	*p* value^a^
Age, mean (SD), year	60.96 (12.70)	64.66 (11.70)	<0.001*	64.48 (11.62)	64.29 (11.41)	0.784
Male, no. (%)	198 (19.1)	114 (19.6)	0.880	105 (19.0)	103 (18.6)	0.939
BMI, kg/m2, mean (SD)	21.91 (2.63)	22.01 (2.44)	0.426	21.93 (2.52)	21.99 (2.45)	0.673
BMI categories, no. (%)			0.670			0.768
Normal (<18.5)	766 (74.5)	435 (75.3)		416 (75.2)	417 (75.4)	
Underweight [18.5, 24)	18 (1.8)	11 (1.9)		7 (1.3)	11 (2.0)	
Overweight [24–28)	173 (16.8)	101 (17.5)		95 (17.2)	94 (17.0)	
Obese (≥28)	71 (6.9)	31 (5.4)		35 (6.3)	31 (5.6)	
Family history of RI-related, no. (%)	56 (5.4)	16 (2.7)	0.018*	16 (2.9)	13 (2.4)	0.707
Operation history of RI-related, no. (%)	82 (7.9)	49 (8.4)	0.801	45 (8.1)	45 (8.1)	1.000
Smoker, no. (%)	10 (1.0)	12 (2.1)	0.110	4 (0.7)	6 (1.1)	0.751
Drinking status, no. (%)			0.050*			0.777
Nondrinker	1022 (98.6)	565 (96.9)		548 (99.1)	546 (98.7)	
Ex-drinker	4 (0.4)	4 (0.7)		2 (0.4)	2 (0.4)	
Drinker	10 (1.0)	14 (2.4)		3 (0.5)	5 (0.9)	
Comorbidities						
At least one, no. (%)	162 (15.8)	101 (17.4)	0.440	95 (17.2)	94 (17.0)	1.000
Categories, median (range)	0 (0, 5)	0 (0, 4)	0.327	0 (0, 5)	0 (0, 4)	0.974
Hypertension, no. (%)	109 (10.6)	69 (11.9)	0.486	73 (13.2)	64 (11.6)	0.465
Diabetes mellitus, no. (%)	27 (2.6)	18 (3.1)	0.695	18 (3.3)	20 (3.6)	0.869
Duration of RA, median (IQR), year	6.83 (3.00, 12.83)	6.50 (2.33, 11.96)	0.162	6.08 (2.92, 12.08)	6.58 (2.42, 11.92)	0.842

PSM, propensity score matching; PP, per-protocol; IM, integrative medicine; WM, western medicine; SD, standard deviation; IQR, interquartile range; BMI, body mass index; RI, rheumatic immunity; RA, rheumatoid arthritis.

a *p* values are calculated by Variance Analysis, Chi-square test, or Kruskal Wallis test as appropriate.

**FIGURE 2:**
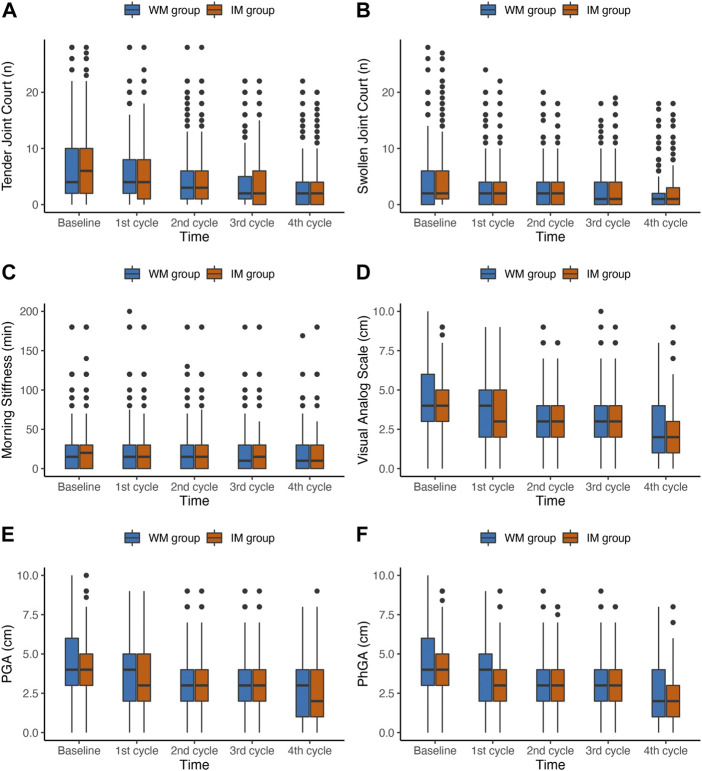
Box plots of changes in joint, morning stiffness, and pain outcomes during the first year of follow-up in PP population. IM: Integrative medicine, WM: Western medicine, PGA, PhGA, patient’s and doctor’s global assessment of disease activity based on visual analogue scale.

Changes in clinical manifestations measures of RA in the ITT and PP population from baseline to 4 follow-up visits were shown in Supplementary Table S5 and Table 3, respectively. The comparison in different rheumatoid arthritis clinical manifestations between baseline and visit 4 in medication changed group was shown in Supplementary Table S6. In the ITT population, the time × group interaction for all outcomes was not significant (*p* > 0.05). Figure 2 shows the changes of outcomes in six domains related to RA, including joint, morning stiffness, and pain, between baseline and the fourth follow-up, and overall decreased in both IM and WM group in PP population. The results indicated a significant time × group interaction for MS (*p* = 0.049), PGA (*p* = 0.049), and PhGA (*p* = 0.047), indicating that the scores for these three domains in the 2 groups had different trends over the 5 time points. Compared with the WM, the IM significantly decreased the levels of VAS, PGA and PhGA in the PP analysis (VAS: odds ratio (OR), 0.76; 95%CI, 0.63–0.92; *p* = 0.004; PGA: OR, 0.76; 95% CI, 0.64–0.92; *p* = 0.007; and PhGA: OR, 0.77; 95% CI, 0.64, 0.93; *p* = 0.004).

**TABLE 3:** Change in different rheumatoid arthritis clinical manifestations at different visits in PP population, median (IQR) unless otherwise stated.

	Baseline		Visit 1		Visit 2		Visit 3		Visit 4		Group	Time	Group: Time
	IM	WM	IM	WM	IM	WM	IM	WM	IM	WM			
TJC (n)	6 (2, 10)	4 (2, 10)	4 (1, 8)	4 (2, 8)	3 (1, 6)	3 (1, 6)	2 (0, 6)	2 (1, 5)	2 (0, 4)	2 (0, 4)	0.293	<0.001^a^	0.623
SJC (n)	2 (1, 6)	2 (0, 6)	2 (0, 4)	2 (0, 4)	2 (0, 4)	2 (0, 4)	1 (0, 4)	1 (0, 4)	1 (0, 3)	1 (0, 2)	0.437	<0.001^a^	0.438
MS (cm)	20 (0, 30)	15 (0, 30)	15 (0, 30)	15 (0, 30)	15 (0, 30)	15 (0, 30)	15 (0, 30)	10 (0, 30)	10 (0, 30)	10 (0, 30)	0.192	<0.001^a^	0.049^a^
VAS (cm)	4 (3, 5)	4 (3, 6)	3 (2, 5)	4 (2, 5)	3 (2, 4)	3 (2, 4)	3 (2, 4)	3 (2, 4)	2 (1, 3)	2 (1, 4)	0.004^a^	<0.001^a^	0.065
PGA (cm)	4 (3, 5)	4 (3, 6)	3 (2, 5)	4 (2, 5)	3 (2, 4)	3 (2, 4)	3 (2, 4)	3 (2, 4)	2 (1, 4)	3 (1, 4)	0.007^a^	<0.001^a^	0.049^a^
PhGA (cm)	4 (3, 5)	4 (3, 6)	3 (2, 4)	4 (2, 5)	3 (2, 4)	3 (2, 4)	3 (2, 4)	3 (2, 4)	2 (1, 3)	2 (1, 4)	0.004^a^	<0.001^a^	0.047^a^
ESR (mg/h)	25 (15, 40)	27 (14, 45)	24.00 (14.00, 36.75)	26.50 (16.00, 40.25)	25 (15, 38)	25 (14, 40)	23.00 (13.00, 35.00)	22.00 (13.00, 38.25)	22 (15.00, 34.00)	23.00 (13.00, 37.00)	0.238	0.003	0.140
CRP (mg/L)	2.72 (0.83, 9.31)	4.24 (1.06, 12.94)	1.98 (0.56, 9.47)	3.45 (1.11, 11.26)	2.02 (0.62, 8.34)	3.28 (0.90, 9.90)	2.32 (0.58, 8.00)	2.80 (0.60, 10.05)	2.16 (0.64, 7.47)	2.84 (0.62, 8.67)	0.164	0.003	0.188
RF (IU/ml)	96.30 (24.00, 195.00)	105. (24.61, 270.75)	94.0 (28.90, 151.50)	102.0 (37.5, 240.6)	68.50 (29.9, 177.8)	102.0 (39.5, 185.3)	50.0 (20.0, 116.0)	82.0 (30.0, 168.0)	50.9 (22.4, 119.0)	51.90 (20.0, 117.0)	0.694	0.003	0.763
Anti-CCP (RU/ml)	153.50 (42.25, 371.00)	146 (32.95, 424.45)	164.50 (31.0, 501.0)	37.00 (25.50, 194.17)	63.0 (26.50, 204.50)	73.8 (32.08, 344.0)	145.00 (25.00, 210.00)	183.0 (25.00, 258.0)	176.5 (56.9, 228.5)	131.0 (22.44, 375.50)	0.510	0.707	0.432
DAS28	4.03 (3.23, 4.85)	3.94 (3.08, 4.96)	3.72 (2.72, 4.48)	3.82 (2.76, 4.76)	3.56 (2.55, 4.44)	3.58 (2.56, 4.42)	3.31 (2.40, 4.23)	3.41 (2.35, 4.33)	3.21 (2.29, 4.01)	3.23 (2.16, 4.13)	0.030^a^	<0.001^a^	0.163
SDAI	21.92 (12.84, 36.00)	23.68 (11.91, 41.60)	16.50 (8.98, 30.12)	19.46 (9.75, 33.87)	15.56 (8.01, 27.23)	17.54 (8.88, 29.00)	12.84 (7.22, 24.04)	14.50 (6.94, 27.91)	12.50 (6.50, 22.38)	13.70 (5.89, 24.52)	0.090	<0.001^a^	0.145
CDAI	17.00 (10.00, 25.00)	16.00 (9.00, 26.00)	13.00 (7.00, 20.00)	14.00 (7.00, 22.00)	11.00 (6.00, 18.00)	12.00 (6.00, 18.00)	10.00 (5.00, 16.00)	10.00 (5.00, 16.00)	8.00 (4.00, 14.00)	8.00 (4.00, 14.00)	0.074	<0.001^a^	0.207
HAQ	0.30 (0.15, 0.50)	0.25 (0.10, 0.50)	0.25 (0.10, 0.45)	0.25 (0.10, 0.45)	0.25 (0.10, 0.45)	0.25 (0.10, 0.45)	0.25 (0.10, 0.40)	0.25 (0.05, 0.45)	0.20 (0.05, 0.40)	0.20 (0.05, 0.40)	0.238	<0.001^a^	0.425

PP, per-protocol; IM, integrative medicine; WM, western medicine; IQR, interquartile range; TJC, tender joint court; SJC, swollen joint count; MS, morning stiffness; VAS, visual analog scale; PGA, patient’s global assessment of disease activity; PhGA, physician’s global assessment of disease activity; ESR, erythrocyte sedimentation rate; CRP, c-reaction protein; RF, rheumatoid factor; Anti-CCP, anti-cyclic citrullinated peptide; DAS28, disease activity score 28; SDAI, simplified disease activity index; CDAI, clinical disease activity index; HAQ, health assessment questionnaire.

a Significant at 0.05.

**FIGURE 3:**
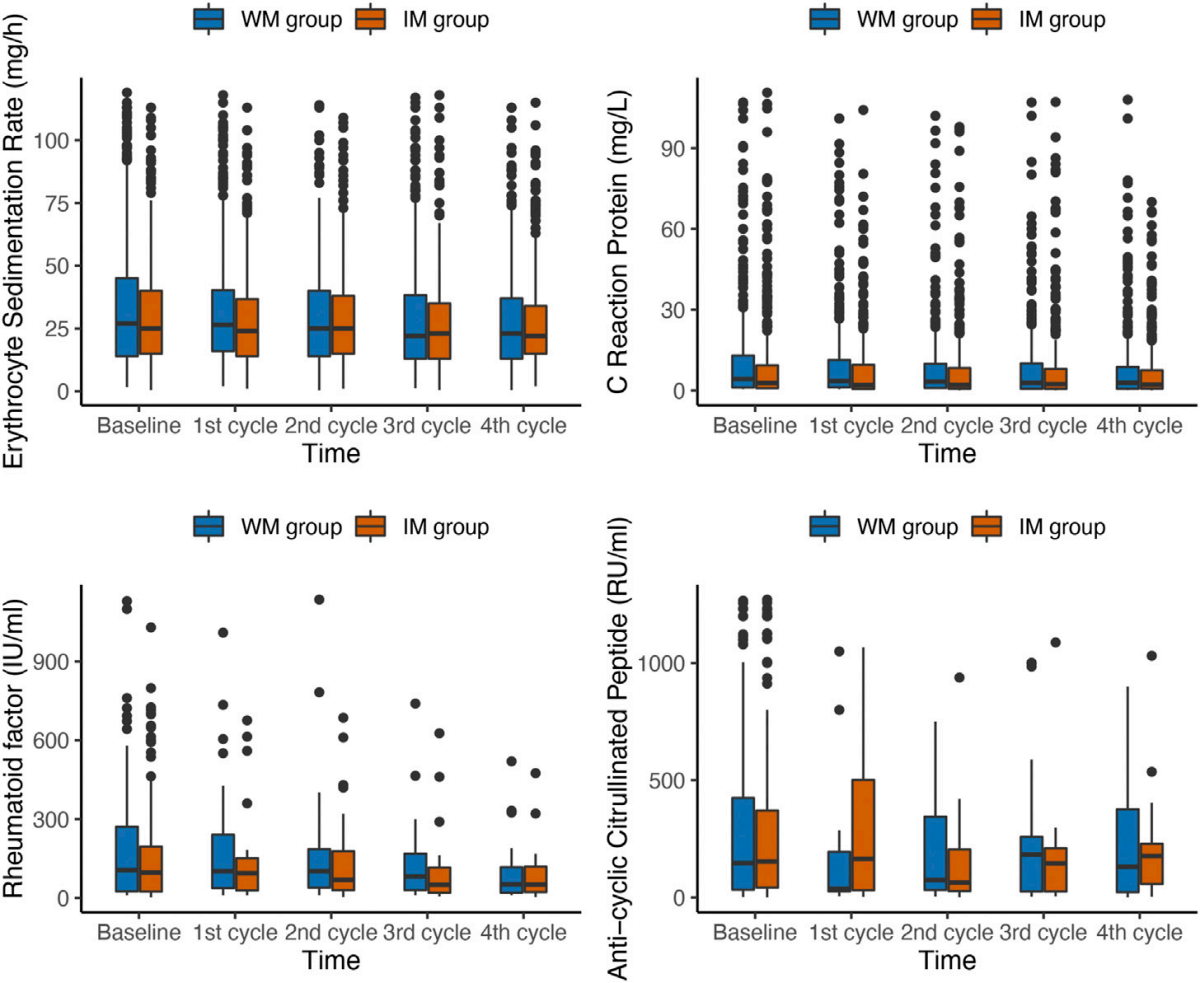
Box plots of changes in laboratory outcomes during the first year of follow-up in PP population. IM: Integrative medicine, WM: Western medicine, PGA, PhGA, patient’s and doctor’s global assessment of disease activity based on visual analogue scale.

The variation patterns of laboratory outcomes in the IM and WM groups in PP population at baseline and follow-up were shown in Figure 3. The average level of RA related laboratory indicators in each domain in both two groups gradually decreased over time (Table 3). The result indicated a significant time × group interaction for ESR (*p* = 0.032). The ESR level of patients in the IM and WM groups decreased gradually from baseline 28 mm/h to 21 and 22 mm/h, respectively. However, the level of CRP, RF and CCP reduced by IM was not significantly higher than that of WM (*p* > 0.05).

**FIGURE 4:**
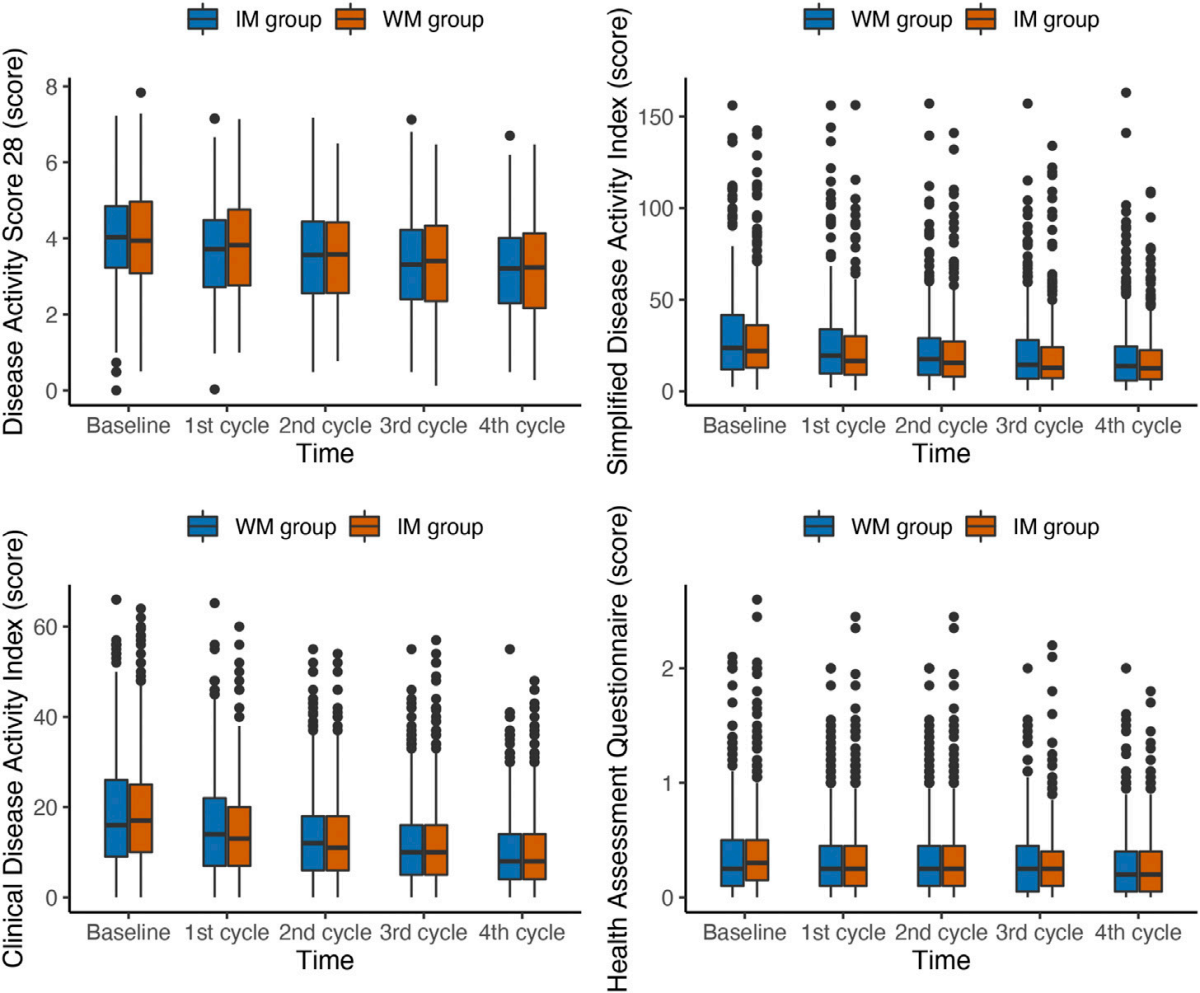
Box plots of changes in Composite outcomes during the first year of follow-up in PP population. IM: Integrative medicine, WM: Western medicine, PGA, PhGA, patient’s and doctor’s global assessment of disease activity based on visual analogue scale.

In the PP population, the scores of DAS28, SDAI, CDAI and HAQ from patients were gradually reduced from baseline among groups over time in 1 year (Figure 4). The median DAS28 score decreased from 4.07 at baseline to 3.21 at the fourth follow-up in the IM group and from 3.94 to 3.23 in the WM. The GEE analysis with PSM showed that the IM was more beneficial to significantly reduce DAS28 in the PP population (OR, 0.84; 95% CI, 0.73–0.98; *p* = 0.030; Table 3). Both IM and WM were beneficial to reduce the scores of SDAI, CDAI and HAQ, and there was no statistical significance between the two groups (*p* > 0.05).

## 4 Discussion

This is the first study compared the use of IM and WM for treatment of RA in real-world clinical settings with proper design, participants in the study had been followed-up for months with continuous collection of variables through blood tests, interviews, and questionnaires. The analysis with matching showed that IM could significantly reduce levels of VAS, PGA, PhGA, and DAS28 in the population who received the same medication at baseline and at follow-up.

A possible explanation for the finding is that the application of IM allows physicians to have further treatment options for alleviating global symptoms. Under the primary health-care system in mainland China, the diversity of WM drugs provided in hospitals is limited, and Chinese herbal medicine can be used as complementary approach. Chinese herbal medicine not only can be applied to treat RA-related symptoms, but also help to relief adverse effects caused by the WM (Xing et al., 2020). In this study, however, we were not able to specify the potential adverse effects which could be avoided by the use of IM.

Biologically, the involvement of Chinese herbal medicine induces multiple treatment pathways and mechanisms especially single herb can contain various kinds of active ingredients targeting RA-related receptors and biomarkers (Chen et al., 2004; van der Greef et al., 2010; Seca and Franconi, 2018). With human clinical trials and animal studies suggested the efficacy and safety of IM with Chinese herbal medicine and their extractions (Liu et al., 2018; Wang et al., 2019), IM approaches are expected to motivate improvements in RA treatments strategies and outcomes. Moreover, as biological agents for rheumatoid arthritis are gradually under consideration for national health insurance coverage, we look forward to investigating the role of biological agents in IM treatment strategies in future studies.

Prevalence of RA is estimated to be 0.2–0.3% in China with approximately 3-million patients (Zeng et al., 2008). Data provided by this perspective cohort will play a key role in reflection of the health service in China by revealing the medical treatments given to the RA patients. In recent years, IM approaches have been receiving attention globally not only among the patients, but also among physicians, researchers, and decision-makers. However, in many diseases, the benefits brought by IM over WM are still unclear. As a result, we hope our analysis could facilitate other teams/nations to carry out further IM-related research for RA in particular through clinical trials with investigation of underlying treatment mechanisms, and eventually, facilitate development of corresponding IM clinical practice guidelines.

Our study has strengths in large sample size, months of follow-up period, high response rate, high compliance of participants, and the study design. With interviews and medical visits to provide complete and accurate information, patients were expected to be relatively easy to follow over time and more likely to maintain participation in long-term. Aimed to provide an overview of IM and WM treatments in RA patients, this study is unique with generalizability as covered patients in multiple provinces. In limitations, dosages and formula of the medicines and disease stages were not involved in the analysis. Then, adverse events were not clearly identified and analyzed due to limitations in medical records and data collection. Finally, residual confounding cannot be ignored in cohort studies due to incomplete control of confounders.

## 5 Conclusion

This study suggests that compare to WM, IM has advantages in improving RA-related outcomes. However, the statistical significance might not reveal significant clinical difference. Further studies should be focused on specific treatment strategies and/or disease stages.

## Data Availability

The original contributions presented in the study are included in the article/Supplementary Material, further inquiries can be directed to the corresponding authors.

## Abbreviations

RA, Rheumatoid arthritis; IM, Integrative medicine; CM, Chinese medicine; WM, Western medicine; NSAIDs, non-steroidal anti-inflammatory drugs; DMARDs, disease-modifying antirheumatic drugs; ACR, American College of Rheumatology; EULAR, European Alliance of Associations for Rheumatology; ITT, intention-to-treat; PP, per-protocol; BMI, body mass index; RI, rheumatic immunity; PSM, propensity score matching; DAS28, disease activity score 28; TJC, tender joint count; SJC, swollen joint count; MS, morning stiffness; VAS, visual analog scale; PGA, patient’s global assessment of disease activity; PhGA, physician’s global assessment of disease activity; ESR, erythrocyte sedimentation rate; CRP, c-reactive protein; RF, rheumatoid factor; Anti-CCP, anti-cyclic citrullinated peptide; SDAI, simplified disease activity index; CDAI, clinical disease activity index; HAQ, health assessment questionnaire; MI, multiple imputation.
