# Supplementary material for: The combination of Chinese and Western Medicine in the management of rheumatoid arthritis: A real-world cohort study across China
Source: Front Pharmacol. 2022 Oct 6;13:933519. doi: 10.3389/fphar.2022.933519 (PMC9582451; doi:10.3389/fphar.2022.933519)
Supplement: Supplementary file 1 [file Table1.DOCX]

**Supplementary Table I. Baseline characteristic of the lost group and follow up group.**

| **Demographics** | **Lost group**  **(n=996)** | **Follow up group**  **(n=2199)** | ***P* value^†^** |
| --- | --- | --- | --- |
| **Age, median (IQR), year** | 59.00 (51.00, 67.00) | 63.00 (55.00, 70.00) | <0.001* |
| **Male, no. (%)** | 171 (17.2) | 412 (18.7) | 0.311 |
| **BMI, kg/m2, median (IQR)** | 21.87 (20.13, 23.88) | 21.64 (20.20, 23.43) | 0.087 |
| **BMI categories, no. (%)** |  |  | <0.001* |
| **Normal (<18.5)** | 656 (66.7) | 1619 (74.1) |  |
| **Underweight [18.5, 24)** | 31 (3.2) | 41 (1.9) |  |
| **Overweight [24-28)** | 204 (20.7) | 383 (17.5) |  |
| **Obese (>=28)** | 93 (9.5) | 141 (6.5) |  |
| **Family history of RI-related, no. (%)** | 61 (6.1) | 112 (5.1) | 0.268 |
| **Operation history of RI-related, no. (%)** | 79 (7.9) | 173 (7.9) | 1.000 |
| **Smoker, no. (%)** | 31 (3.1) | 35 (1.6) | 0.008* |
| **Drinking status, no. (%)** |  |  | 0.544 |
| **Nondrinker** | 968 (97.2) | 2150 (97.8) |  |
| **Ex-drinker** | 10 (1.0) | 15 (0.7) |  |
| **Drinker** | 18 (1.8) | 34 (1.5) |  |
| **Comorbidities** |  |  |  |
| **At least one, no. (%)** | 195 (19.9) | 338 (15.5) | 0.003* |
| **Categories, median (range)** | 0 (0, 7) | 0 (0, 5) | 0.001* |
| **Hypertension, no. (%)** | 94 (9.6) | 227 (10.4) | 0.518 |
| **Diabetes mellitus, no. (%)** | 17 (1.7) | 60 (2.8) | 0.111 |
| **Duration of RA, median (IQR), year** | 5.25 (2.08, 11.58) | 6.17 (2.42, 12.17) | 0.018* |

SD, Standard Deviation; IQR, interquartile range; BMI, body mass index; RI, Rheumatic immunity; RA, rheumatoid arthritis.

†P values are calculated by Variance Analysis, Chi-square test, or Kruskal Wallis test as appropriate. *Significant at 0.05.
