# Supplementary material for: The combination of Chinese and Western Medicine in the management of rheumatoid arthritis: A real-world cohort study across China
Source: Front Pharmacol. 2022 Oct 6;13:933519. doi: 10.3389/fphar.2022.933519 (PMC9582451; doi:10.3389/fphar.2022.933519)
Supplement: Supplementary file 2 [file Table2.DOCX]

**Supplementary Table II. Baseline characteristic of the unchanged medication group and medication changed group.**

| **Demographics** | **Unchanged medication group**  **(n=1619)** | **Medication changed group**  **(n=1576)** | ***P* value^†^** |
| --- | --- | --- | --- |
| **Age, median (IQR)** | 63.00 (55.00, 70.00) | 61.00 (52.00, 68.00) | <0.001* |
| **Male, no. (%)** | 312 (19.3) | 271 (17.2) | 0.141 |
| **BMI, kg/m^2^, median (IQR)** | 21.71 (20.28, 23.34) | 21.72 (20.06, 23.73) | 0.522 |
| **BMI categories, no. (%)** |  |  | 0.002* |
| **Normal (<18.5)** | 1212 (74.9) | 1086 (68.9) |  |
| **Underweight [18.5, 24)** | 103 (6.4) | 132 (8.4) |  |
| **Overweight [24-28)** | 274 (16.9) | 315 (20.0) |  |
| **Obese (>=28)** | 30 (1.9) | 43 (2.7) |  |
| **Family history of RI-related, no. (%)** |  |  | 0.018* |
| **No** | 1547 (95.6) | 1475 (93.6) |  |
| **Yes** | 72 (4.4) | 101 (6.4) |  |
| **Operation history of RI-related, no. (%)** |  |  | 0.713 |
| **No** | 1488 (91.9) | 1455 (92.3) |  |
| **Yes** | 131 (8.1) | 121 (7.7) |  |
| **Smoker, no. (%)** |  |  | 0.006* |
| **No** | 1597 (98.6) | 1532 (97.2) |  |
| **Yes** | 22 (1.4) | 44 (2.8) |  |
| **Drinking status, no. (%)** |  |  | 0.137 |
| **Nondrinker** | 1587 (98.0) | 1531 (97.1) |  |
| **Ex-drinker** | 8 (0.5) | 17 (1.1) |  |
| **Drinker** | 24 (1.5) | 28 (1.8) |  |
| **At least one comorbidity, no. (%)** |  |  | 0.471 |
| **No** | 1352 (83.5) | 1300 (82.5) |  |
| **Yes** | 267 (16.5) | 276 (17.5) |  |
| **Categories of comorbidity, median (range)** | 0.00 (0, 5) | 0.00 (0, 7) | 0.284 |
| **Hypertension, no. (%)** |  |  | 0.056* |
| **No** | 1438 (88.8) | 1433 (90.9) |  |
| **Yes** | 181 (11.2) | 143 (9.1) |  |
| **Diabete, no. (%)** |  |  | 0.261 |
| **No** | 1573 (97.2) | 1542 (97.8) |  |
| **Yes** | 46 (2.8) | 34 (2.2) |  |
| **Duration of RA, median (IQR), year** | 6.58 (2.75, 12.50) | 5.25 (2.08, 11.25) | <0.001* |

SD, Standard Deviation; IQR, interquartile range; BMI, body mass index; RI, Rheumatic immunity; RA, rheumatoid arthritis.

†P values are calculated by Variance Analysis, Chi-square test, or Kruskal Wallis test as appropriate. *Significant at 0.05.
