# Supplementary material for: The combination of Chinese and Western Medicine in the management of rheumatoid arthritis: A real-world cohort study across China
Source: Front Pharmacol. 2022 Oct 6;13:933519. doi: 10.3389/fphar.2022.933519 (PMC9582451; doi:10.3389/fphar.2022.933519)
Supplement: Supplementary file 3 [file Table5.DOCX]

**Supplementary Table V. Change in different rheumatoid arthritis clinical manifestations at different visits in ITT Population, median (IQR) unless otherwise stated.**

| **Outcomes** | **Baseline** | | **Visit 1** | | **Visit 2** | | **Visit 3** | | **Visit 4** | |  |  | **Group : Time** |
| --- | --- | --- | --- | --- | --- | --- | --- | --- | --- | --- | --- | --- | --- |
|  | **IM** | **WM** | **IM** | **WM** | **IM** | **WM** | **IM** | **WM** | **IM** | **WM** | **Group** | **Time** |  |
| **TJC (n)** | 6 (2, 10) | 5 (2, 10) | 4 (1, 8) | 4 (1, 8) | 3 (1, 6) | 3 (1, 6) | 2 (1, 6) | 2 (0, 6) | 2 (0, 4) | 2 (0, 4) | 0.585 | <0.001* | 0.809 |
| **SJC (n)** | 2 (1, 6) | 2 (0, 6) | 2 (0, 5) | 2 (0, 4) | 2 (0, 4) | 2 (0, 4) | 1 (0, 4) | 1 (0, 4) | 1 (0, 3) | 1 (0, 3) | 0.470 | <0.001* | 0.275 |
| **MS (cm)** | 20 (0, 40) | 30 (2, 60) | 20 (0, 30) | 20 (5, 30) | 20 (0, 30) | 20 (0, 30) | 15 (0, 30) | 15 (0, 30) | 15 (0, 30) | 10 (0, 30) | 0.428 | <0.001* | 0.059 |
| **VAS (cm)** | 4 (3, 6) | 4 (3, 6) | 4 (2, 5) | 4 (2, 5) | 3 (2, 4) | 3 (2, 4) | 3 (2, 4) | 3 (2, 4) | 2 (1, 3) | 2 (1, 4) | 0.243 | <0.001* | 0.313 |
| **PGA (cm)** | 4 (3, 5.5) | 4 (3, 6) | 3.5 (2, 5) | 4 (2, 5) | 3 (2, 4) | 3 (2, 4) | 3 (2, 4) | 3 (2, 4) | 2 (1, 3) | 2 (1, 3) | 0.347 | <0.001* | 0.446 |
| **PhGA (cm)** | 4 (3, 6) | 4 (3, 6) | 4 (2, 5) | 4 (2, 5) | 3 (2, 4) | 3 (2, 4) | 3 (2, 4) | 3 (2, 4) | 2 (1, 4) | 3 (1, 4) | 0.140 | <0.001* | 0.230 |
| **ESR (mg/h)** | 28.00 (15.00, 48.00) | 27.00 (15.00, 45.00) | 26.00 (15.00, 44.00) | 25.00 (15.00, 38.00) | 25.00 (15.00, 41.00) | 24.00 (14.00, 39.00) | 24.00 (14.00, 40.00) | 23.00 (14.00, 38.00) | 23.00 (12.00, 38.00) | 22.00 (14.00, 34.00) | 0.232 | <0.001* | 0.355 |
| **CRP (mg/L)** | 4.42 (1.06, 16.03) | 3.55 (0.90, 14.42) | 3.51 (1.00, 11.77) | 2.48 (0.69, 10.50) | 3.62 (0.97, 11.95) | 2.54 (0.68, 10.51) | 3.33 (0.68, 10.38) | 2.55 (0.64, 8.90) | 2.59 (0.60, 8.89) | 1.97 (0.62, 7.05) | 0.363 | <0.003 | 0.789 |
| **RF (IU/ml)** | 107.00 (24.37, 271.75) | 107.0 (31.0, 219.30) | 90.4 (28.05, 256.58) | 102.0 (37.05, 236.1) | 64.75 (26.2, 198.50) | 64.50 (20.0, 165.60) | 67.35 (21.0, 184.55) | 70.0 (25.0, 188.0) | 78.0 (20.0, 133.0) | 53.5 (23.12, 125.25) | 0.116 | 0.017* | 0.140 |
| **Anti-CCP (RU/ml)** | 254.95 (45.8, 1009.02) | 206.0 (45.0, 733.99) | 174.0 (29.50, 506.0) | 219.0 (31.75, 888.8) | 189.80 (35.6, 712.0) | 100.0 (32.0, 407.50) | 175.0 (27.60, 339.50) | 298.0 (110, 1025.9) | 109.70 (24.50, 466.0) | 190.0 (77.0, 794.0) | 0.484 | 0.484 | 0.993 |
| **DAS28** | 4.05 (3.11, 5.07) | 4.09 (3.24, 5.04) | 3.69 (2.71, 4.60) | 3.77 (2.76, 4.59) | 3.60 (2.60, 4.39) | 3.56 (2.64, 4.39) | 3.41 (2.39, 4.36) | 3.33 (2.46, 4.23) | 3.15 (2.24, 4.11) | 3.18 (2.24, 4.00) | 0.470 | <0.001* | 0.866 |
| **SDAI** | 25.62 (13.56, 44.72) | 24.50 (14.25, 44.71) | 19.60 (10.04, 32.91) | 18.72 (9.80, 32.92) | 18.52 (10.07, 30.02) | 17.06 (8.64, 30.00) | 15.43 (8.05, 28.50) | 14.50 (7.50, 26.01) | 13.50 (6.50, 23.77) | 11.95 (5.98, 22.05) | 0.590 | <0.001* | 0.825 |
| **CDAI** | 17.00 (10.00, 27.00) | 17.00 (10.00, 28.00) | 13.00 (7.00, 22.00) | 14.00 (8.00, 21.00) | 12.00 (6.00, 18.00) | 12.00 (6.00, 18.00) | 10.00 (5.00, 16.00) | 10.00 (5.00, 16.00) | 8.00 (4.00, 14.75) | 8.00 (4.00, 14.00) | 0.652 | <0.001* | 0.916 |
| **HAQ** | 0.3 (0.1, 0.59) | 0.25 (0.1, 0.55) | 0.25 (0.05, 0.45) | 0.25 (0.05, 0.5) | 0.25 (0.05, 0.45) | 0.25 (0.05, 0.5) | 0.25 (0.05, 0.4) | 0.25 (0.05, 0.45) | 0.2 (0.05, 0.4) | 0.25 (0.05, 0.45) | 0.718 | <0.001* | 0.369 |

ITT, Intention-To-Treat; IM, Integrative medicine; WM, Western medicine; IQR, Interquartile range; TJC, Tender joint court; SJC, Swollen joint count; MS, Morning stiffness; VAS, visual analog scale ; PGA, PhGA, patient's and doctor's global assessment of disease activity based on visual analogue scale; ESR, erythrocyte sedimentation rate; CRP, C reaction protein; RF, Rheumatoid factor; Anti-CCP, Anti-cyclic citrullinated peptide; DAS28, disease activity score 28; SDAI, simplified disease activity index; CDAI, clinical disease activity index; HAQ, health assessment questionnaire.

*Significant at 0.05.
