# Supplementary material for: The combination of Chinese and Western Medicine in the management of rheumatoid arthritis: A real-world cohort study across China
Source: Front Pharmacol. 2022 Oct 6;13:933519. doi: 10.3389/fphar.2022.933519 (PMC9582451; doi:10.3389/fphar.2022.933519)
Supplement: Supplementary file 4 [file Table3.DOCX]

**Supplementary Table III. Number of available and missing values.**

| **Characteristics** | **Total N available** | **Missing, n (%)** |
| --- | --- | --- |
| **Age** | 3157 | 38 (1.19) |
| **Gender** | 3195 | 0 |
| **BMI** | 3168 | 27 (0.85) |
| **Family history of RI-related** | 3195 | 0 |
| **Operation history of RI-related** | 3195 | 0 |
| **Smoking status** | 3195 | 0 |
| **Drinking status** | 3195 | 0 |
| **Comorbidities** | 3159 | 36 (1.13) |
| **Hypertension** | 3159 | 36 (1.13) |
| **Diabetes mellitus** | 3159 | 36 (1.13) |
| **Duration of RA** | 3193 | 2 (0.06) |

BMI, body mass index; RI, Rheumatic immunity; RA, rheumatoid arthritis.
