# Supplementary material for: The combination of Chinese and Western Medicine in the management of rheumatoid arthritis: A real-world cohort study across China
Source: Front Pharmacol. 2022 Oct 6;13:933519. doi: 10.3389/fphar.2022.933519 (PMC9582451; doi:10.3389/fphar.2022.933519)
Supplement: Supplementary file 5 [file Table4.DOCX]

**Supplementary Table IV. Patient Characteristics Before and After PSM in ITT Population.**

| **Characteristics** | **Unmatched (N=3195)** | | | **After PSM (N=2662)** | | |
| --- | --- | --- | --- | --- | --- | --- |
|  | **WM (n=1816)** | **IM (n=1379)** | ***P* value^†^** | **WM (n=1331)** | **IM (n=1331)** | ***P* value^†^** |
| **Age, year, mean (SD)** | 60.34 (12.82) | 62.02 (12.59) | <0.001* | 62.07 (12.55) | 61.78 (12.44) | 0.558 |
| **Male, n (%)** | 321 (17.7) | 262 (19.0) | 0.361 | 241 (18.1) | 246 (18.5) | 0.841 |
| **BMI, kg/m^2^, mean (SD)** | 21.89 (2.66) | 22.09 (2.79) | 0.036* | 22.01 (2.65) | 22.03 (2.66) | 0.812 |
| **BMI categories, n (%)** |  |  | 0.187 |  |  | 0.488 |
| **Normal (<18.5)** | 1329 (73.2) | 969 (70.3) |  | 976 (73.3) | 942 (70.8) |  |
| **Underweight [18.5, 24)** | 134 (7.4) | 101 (7.3) |  | 87 (6.5) | 97 (7.3) |  |
| **Overweight [24-28)** | 317 (17.5) | 272 (19.7) |  | 243 (18.3) | 261 (19.6) |  |
| **Obese (>=28)** | 36 (2.0) | 37 (2.7) |  | 25 (1.9) | 31 (2.3) |  |
| **Family history of RI-related, n (%)** | 100 (5.5) | 73 (5.3) | 0.854 | 71 (5.3) | 67 (5.0) | 0.793 |
| **Operation history of RI-related, n (%)** | 143 (7.9) | 109 (7.9) | 1.000 | 107 (8.0) | 104 (7.8) | 0.886 |
| **Smoker, n (%)** | 28 (1.5) | 38 (2.8) | 0.024* | 26 (2.0) | 28 (2.1) | 0.891 |
| **Drinking status, n (%)** |  |  | 0.022* |  |  | 0.856 |
| **Nondrinker** | 1784 (98.2) | 1334 (96.7) |  | 1304 (98.0) | 1300 (97.7) |  |
| **Ex-drinker** | 11 (0.6) | 14 (1.0) |  | 9 (0.7) | 11 (0.8) |  |
| **Drinker** | 21 (1.2) | 31 (2.2) |  | 18 (1.4) | 20 (1.5) |  |
| **Comorbidities** |  |  |  |  |  |  |
| **At least one, n (%)** | 287 (15.8) | 256 (18.6) | 0.044* | 233 (17.5) | 228 (17.1) | 0.838 |
| **Categories, median (range)** | 0 (0, 7) | 0 (0, 7) | 0.020* | 0 (0, 6) | 0 (0, 5) | 0.884 |
| **Hypertension, n (%)** | 183 (10.1) | 141 (10.2) | 0.938 | 137 (10.3) | 137 (10.3) | 1.000 |
| **Diabetes, n (%)** | 41 (2.3) | 39 (2.8) | 0.364 | 37 (2.8) | 36 (2.7) | 1.000 |
| **Duration of RA, year, median (IQR)** | 6 (2.58, 12.3) | 6.08 (2.08, 11.8) | 0.187 | 5.83 (2.33, 12.1) | 6.08 (2.08, 11.8) | 0.534 |

PSM, Propensity Score Matching; IM, Integrative medicine; WM, Western medicine; SD, Standard Deviation; IQR, interquartile range; BMI, body mass index; RI, Rheumatic immunity; RA, rheumatoid arthritis.

†P values are calculated by Variance Analysis, Chi-square test, or Kruskal Wallis test as appropriate.
