# Supplementary material for: The combination of Chinese and Western Medicine in the management of rheumatoid arthritis: A real-world cohort study across China
Source: Front Pharmacol. 2022 Oct 6;13:933519. doi: 10.3389/fphar.2022.933519 (PMC9582451; doi:10.3389/fphar.2022.933519)
Supplement: Supplementary file 6 [file Table6.DOCX]

**Supplementary Table VI. Comparison in different rheumatoid arthritis clinical manifestations between baseline and visit 4 in medication changed group, median (IQR) unless otherwise stated.**

| **Outcomes** | **Baseline** | **Visit 4** | ***P* value^†^** |
| --- | --- | --- | --- |
| **Tender joint court (n)** | 6 (2, 10.75) | 2 (0, 4) | <0.001* |
| **Swollen joint count (n)** | 3 (1, 7) | 1 (0, 3) | <0.001* |
| **Morning stiffness (cm)** | 30 (5, 60) | 20 (0, 30) | <0.001* |
| **Visual analog scale (cm)** | 4 (3, 6) | 2 (1, 4) | <0.001* |
| **Patient's global assessment (cm)** | 4 (3, 6) | 2 (1, 4) | <0.001* |
| **Doctor's global assessment (cm)** | 5 (3, 6) | 3 (1.38, 4) | <0.001* |
| **Erythrocyte sedimentation rate (mg/h)** | 30 (16, 53) | 21 (12, 36) | <0.001* |
| **C reaction protein (mg/L)** | 5.12 (1.14, 20.46) | 1.85 (0.50, 7.78) | <0.001* |
| **Rheumatoid factor (IU/ml)** | 110.70 (33.50, 235.25) | 53.00 (27.50, 151.00) | 0.210 |
| **Anti-cyclic citrullinated peptide (RU/ml)** | 202.30 (38.00, 661.00) | 109.35 (28.75, 571.50) | 0.549 |
| **Disease activity score 28 (score)** | 4.24 (3.31, 5.23) | 3.11 (2.21, 4.11) | <0.001* |
| **Simplified disease activity index (score)** | 29.14 (15.67, 50.49) | 11.90 (5.61, 23.42) | <0.001* |
| **Clinical disease activity index (score)** | 18.00 (11.00, 29.00) | 8.50 (4.00, 16.00) | <0.001* |
| **Health assessment questionnaire (score)** | 0.25 (0.05, 0.60) | 0.20 (0.04, 0.40) | <0.001* |

†P values are calculated by Kruskal Wallis test. *Significant at 0.05.
